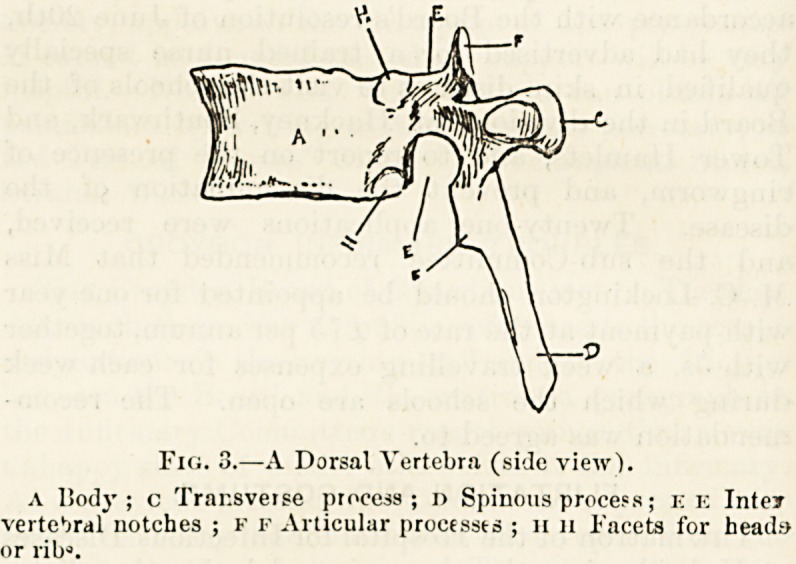# The Hospital. Nursing Section

**Published:** 1901-10-19

**Authors:** 


### The Hospital.

### Nursing Section.

Contributions for this Section of "THE HOSPITAL" should be addressed to the EDITOR, "THE HOSPITAL" NURSING SECTION, 28 & 29 Southampton Street, Strand, London, W.C.

No. 786.—VOL. XXXI. *SATURDAY, OCTOBER* 19, 1901.

### Notes on News from the Nursing World.

### THE COMING CORONATION.

As we anticipated, the possibility of an arrangement being made for "Queen Alexandra's Nurses" to witness the Coronation procession has provoked enthusiastic manifestations of delight. Last week we published a selection from the numerous letters received from members of the Royal National Pension Fund and others expressing their satisfaction at the prospect of being afforded a privilege which they had not expected could be put within their reach; and we have since had a large number of similar communications from all parts of the country. One of our correspondents, whose letters will all be filed for reference, says that she thinks "a group of hundreds of nurses, in indoor uniform, wearing the Queen's brassard, would add materially to the picturesqueness of the scene, and testify to the loyalty of the members of the profession." As to their loyalty, we are sure that no testimony is needed; but we believe that Her Majesty will be gratified if a representative gathering of her nurses can join in the national demonstration on Coronation Day.

### THE WAR NURSES.

THE Director-General of the Army Medical Service informs us that the undermentioned nursing sisters embarked for South Africa in the hospital ship *Simla* on the 5th instant:—Nursing Sister H. T. Young, of the Army Nursing Service; Nursing Sisters B. Roberts, M. Cross, and S. M. Lippiatt, of the Army Nursing Service Reserve; and Misses E. Langley Naylor and G. Ronaldson, civilian nurses. The *Hawarden Castle* arrived at Southampton on the 11th inst., with Nursing Sisters W. M. Pooler and G. Chinnery on board. The *Victorian* came in on Sunday, bringing Nursing Sisters L. L. Watts and E. M. Young. The *Bavarian* arrived at Southampton on Tuesday with Nursing Sisters A. McLeod, Q. Lawrence, C. Hamilton (return to South Africa after leave), L. A. H. Seligman (returns to Natal after leave), and E. H. Hordley on board. All of these return to South Africa after a month's leave.

### SKETCH OF NURSES' UNIFORM BY GENERAL BADEN-POWELL.

OF all the drawings and sketches on view at the Modern Gallery, 175 Bond Street, the most interesting to nurses will be the neat little water-colour by Major-General R. S. S. Baden-Powell. It is by itself on a very good light, lying, in fact, on a ledge under a window; and is lent by the London Sketch Club for exhibition. The sketch represents the design which the General has prepared for the uniform of the Nursing Sisters of the South African Constabulary. It is very simple, and consists of a grey gown, white apron, collar and cuffs, with a shoulder-cape of dark green, trimmed with one line of gold braid, and a tiny corner reveals a red lining. In shape, the cape is similar to the scarlet one worn by the Army Nursing Sisters. The hat is a large broad-brimmed one, dark in colour, and turned up with a bunch of green feathers clasped in a red cross. The nurse wears a buckled green belt, and tan shoes; and her dress clears the ground. Her outdoor cloak, if she has one for cold weather, is not shown. If we wished to criticise the drawing, we should say that, unlike its author, it is a little "timid," but, at any rate, it shows what the General considers a suitable dress for the nurse in South Africa, and that is the main point. The Exhibition will remain open until the 26th instant.

### LOCAL GOVERNMENT BOARD INSPECTORS ON NURSING.

IN another page we publish extracts from the reports of some of the inspectors under the Local Government Board on Poor Law Administration dealing with nursing in workhouse infirmaries. The result of an interesting experiment on the part of the Chichester Guardians is mentioned with satisfaction by Mr. Davy, who says that in his district the general nursing continues to improve. Mr. J. W. Preston says the same, but deplores, as well he may do, the want of attention given to "the necessity of night nursing." Mr. Baldwyn Fleming thinks that the difficulty undoubtedly experienced in obtaining competent nurses for rural workhouses is, "that rural guardians do not make the posts sufficiently desirable to attract good candidates"; and he does not exonerate the workhouse medical officers from blame. Mr. Dansey testifies that the arrangements for nursing in his district are improving; and Mr. Jenner-Fust supplies some useful figures showing how the number of patients per nurse has diminished during the last six years. But it is a matter for regret that more than half of the inspectors are silent, or practically silent, on the subject of nursing. Thus, Mr. Preston Thomas, in his long report on the Western Counties, only mentions the complaint of the superintendent nurse at Exeter; and Mr. Wethered, in seven pages devoted to the West Midland Counties, only remarks that "Nursing is not now done by paupers, but by nurses who are becoming more and more competent." Mr. Lockwood does not mention the word nursing in his report on the Metropolis, and equal reticence is shown by several other inspectors. It seems a pity that they should be so unobservant, or so indifferent, to a question of such recognised importance.

### PROGRESS AND RETROGRESSION IN IRELAND.

IT is very natural that Dr. Cox, as physician to St. Vincent's Hospital, Dublin, should maintain that nuns are the persons best fitted to nurse the sick poor, and in the institution with which he is connected they have rendered invaluable service. But we are glad to see that, apart from other questions, he acknowledges the difficulties of obtaining nuns to do the work everywhere, and entirely approves the action of the Irish Local Government Board in requiring that a trained nurse having charge of a hospital must be one who has resided for not less than two years in a general clinical or other hospital recognised by them, who, after examination, has obtained from such hospital a certificate of proficiency in nursing; whilst as qualified nurse or assistant they recognise any person who, after examination, has obtained a certificate from any public general hospital or workhouse infirmary, fever hospital, or nursing institution recognised by them as an efficient school for medical and surgical nurses. Dr. Cox also thinks that "the zeal and determination" of the Local Government Board is shown by the appointment of Dr. Smith of Naas as an inspector to inquire into the general nursing of the workhouse hospitals. No reasonable person suggests that nursing by religious orders in Ireland should be abruptly done away with. But the course which the Newry Board of Guardians has just resolved to pursue is distinctly reactionary. On the proposal of Mr. Small, who appears to have been prompted by Cardinal Logue, they have passed a resolution to the effect that the infirmaries and the departments connected with them shall be placed in the charge of members of a religious order. They were not unanimous, two of the guardians urging that an advertisement should be issued in the ordinary way, and that applications should not be considered on their religious merits at all, but on the qualifications of the applicants. This protest, however, was treated with scant ceremony, and the nuns of the Convent of Mercy at Newry are apparently to be appointed to perform the duties of the "nurse of the workhouse," as provided for under the regulations of the Local Government Board.

### THE UP-COUNTRY NURSING ASSOCIATION.

The present honorary secretary of the Up-Country Nursing Association for Europeans in India is Mr. Herbert M. Birdwood, who succeeded General Bonus. His address is Dalkeith House, Cambridge Park, Twickenham, and all ordinary inquiries should be addressed to him. But any applications under the rules for the engagement and employment of nurses should be made to Mrs. Sheppard, 10 Chester Place, Regent's Park, N.W., who is always glad to supply copies of the rules and to furnish information about work in India. We may mention that a nurse at the time of her appointment must be over 25 years of age; must have had at least three years' training in a general hospital; and must be prepared to serve for a term of five years.

### A TRAINING SCHOOL AT NEWCASTLE WORKHOUSE HOSPITAL.

The Newcastle-on-Tyne Board of Guardians have very wisely decided, on the recommendation of the hospital committee, to increase the staff of charge nurses from four to six, and to increase their salaries from £30 to £35. They have also determined to increase the staff of probationers from nine to 12, and to undertake the training of probationers. As to the last point, we doubt whether the Board will gain by the innovation. One of the guardians, in commenting on the number of nurses who have left during the last six or seven months, "after a few days' service," said that in his opinion the remedy for such a state of things lay in the separation of the hospital from the workhouse. Until this is done, and a superintendent nurse is invested with absolute power over her staff, the establishment of a training school is premature.

### MORE CHANGES AT THE WOMEN'S HOSPITAL, CORK.

Last week we announced the resignation of Miss Kathleen Disney, the lately appointed lady superintendent of the Women and Children's Hospital at Cork on account of ill health. We now learn that when Miss Disney left Cork on October 2nd she was accompanied by the staff sister and night nurse whom she took with her to Cork last May, and that a third nurse who also joined the staff when Miss Disney was appointed left the hospital several weeks ago. The departure of the lady superintendent was rather sudden, and took place a day or two after meetings of the Ladies' Committee and the Board of Management. We are further informed that one of Miss Baxter's former nurses was telegraphed for and installed as superintendent, while a second was also wired and asked to become a staff nurse. So far, however, there appears to have been no official announcement informing the public of these important changes.

### THE SOCIETY OF TRAINED MASSEUSES.

A copy of the "new prospectus" of the Incorporated Society of Trained Masseuses has been sent to us for notice. The objects of the organisation are set forth in detail, the principal one being to improve the status and training of masseuses," and "to provide for the examination of, and the granting of certificates of qualification to, masseuses." The founders are all trained masseuses, and most of them are well known in the nursing world. A long list of influential members of the medical profession who have signified their approval of the aims and principles of the Society, appears on the first page of the prospectus. It cannot be doubted that their support will be very helpful. Nurses who wish to join can obtain all particulars by writing to the honorary secretary, at the Trained Nurses' Club, 12 Buckingham Street, Strand, W.C.

### CROYDON INFIRMARY TROUBLES.

The Croydon Board of Guardians met on Tuesday. Although there was no public reference to the recent resignations on the nursing staff and to the friction between the Board and the matron, a paragraph in the Infirmary Committee's report proves that a very unhappy state of affairs still exists at the infirmary. At a previous meeting the Committee expressed surprise that they had not received a report from the matron on the stocktaking. The Committee now acknowledged the receipt of a letter from the matron, in reply to the clerk's letter, stating that her instructions from the Board were to take the ward stock and report thereon every three months in January, April, July, and October, but as she was absent on sick leave during the month of April, the stock was not taken, but immediately after her return in May the stock was made up to the standard requirements in accordance with the stocktaker's figures, and "that she had not yet completed the ward stock inventories for the month of July for the reasons given in her report to Dr. Wilson on August 10th, 1901." The Committee received a report from the matron dated August 10th, a copy of which had been furnished by the medical superintendent; and also a letter from the medical superintendent as follows :—

"I did not bring this report under the notice of the Infirmary Committee, as I regarded her excuses as being puerile and insufficient, and a carping criticism on the exceptional arrangements which prevailed during the holiday season. I did not consider her report one with which the Committee should be troubled.—Yours truly,

"Regd. Wilson."

The Committee recommended that the matron be informed that as far as they have been able to ascertain there were no sufficient reasons why the stock-taking of the ward stock in use could not have been taken at the proper time.

### THE NURSES AT THE WESTMINSTER AQUARIUM.

We have been at some pains to ascertain the identity of the "four certificated nurses" who are stated to be in attendance on Madame Christensen, "the fasting woman," at the Westminster Aquarium. One of the four candidly admits that she has had no hospital training, but has been engaged chiefly in maternity cases for years. Another is a German. The two others are employed at a private nursing home in Clapham Road, and according to the superintendent are "fully trained." But she does not seem able to remember where. This lady confesses that the affair is repulsive to her, and thinks that the nurses do not like it. But she affirms that the work of watching Madame Christensen is not more disagreeable than a good many of the things that nurses have to do, and that they go to the Aquarium just as they would go to any other case.

### APPOINTMENT OF A BOARD SCHOOL NURSE.

At the last meeting of the London School Board, the General Purposes Committee reported that, in accordance with the Board's resolution of June 20th, they had advertised for a trained nurse specially qualified in skin diseases to visit the schools of the Board in the divisions of Hackney, Southwark, and Tower Hamlets, and to report on the presence of ringworm, and prevent the dissemination of the disease. Twenty-one applications were received, and the sub-Committee recommended that Miss M. C. Lockington should be appointed for one year with payment at the rate of £75 per annum, together with 5s. a week travelling expenses for each week during which the schools are open. The recommendation was agreed to.

### FLIRTATION AND COSTUME.

The matron of the Hospital for Infectious Diseases at Uxbridge is under the curious delusion that flirtation on the part of nurses can be put down by dressing them in unbecoming costumes. Happily, the members of the Joint Hospital Board declined to act on her suggestion that a "walking-out uniform" of a repellent character should be adopted. The matron has at least one better remedy in her own hands. If she is afraid of any of her staff flirting she should take pains to engage only the most unattractive women—for even nurses are not all visions of grace and beauty. Not that good looks and a disposition to flirt necessarily go together any more than plainness and primness, but the risk of responses on the part of the other sex is less in the latter case. After all, the important point is not that the nurses should act discreetly out of doors, but that they should not flirt on duty to the neglect of their patients.

### INDIFFERENCE AT WARRINGTON.

The prosperity and population of the busy town of Warrington render it something of a scandal that while the modest sum of £550 is needed to maintain efficiently a staff of six trained nurses, the actual amount received by the Warrington District Nursing Association for the year ending June 30 was only £480 18s. 3d. A further amount of £145 was, it is true, subscribed, but it consisted of special donations for furnishing and alterations, therefore the income of the Society did not benefit. As there is only one nurse to rather more than ten thousand of the population, it cannot be alleged that Miss Whitfield, the excellent superintendent, whose suggestion that an invalid kitchen should be established in connection with the Association was approved at the annual meeting, has too large a staff. It is, unfortunately, the same in Warrington as in numerous other places; the few generously support the work, and the many do not contribute a farthing. According to a local authority at least six of the committee of management are non-subscribers. Some of these may be able to render help not less valuable than money; but people who neither give money nor its equivalent, are certainly out of place on the committee of a nursing association.

### A NURSES' JUMBLE SALE.

One of the sources of income of the District Nursing Association of Hammersmith and Fulham is a half-yearly jumble sale, held in spring and autumn, when people are turning out their wardrobes for the coming season. The second this year was held at Carnforth Lodge last week. The customers were mostly very poor women who looked doubtfully at anything marked over a shilling unless it was a good winter dress or cloak, or a coat for husband or son. The "penny bundles" were soon gone, and so were the boots and shoes "Nurse told us it was no good coming unless we were punctual," said one rueful-faced woman who had walked a mile or more because she lost the right 'bus and was too late for anything but leavings. The net result, counted out over tea in the nurses' sitting-room before the evening rounds, including penny entrance fees and "tea-money," exceeded £12 10s.—a very satisfactory sum indeed.

### SHORT ITEMS.

The Queen has forwarded a subscription of five guineas to the Royal Victoria Nursing Home, South Ascot.—Princess Christian has fixed December 12th for the date of her visit to Northampton, when she is to open the nursing institute erected by the town as a memorial to Queen Victoria.—Miss Hetherington, who was for upwards of six years matron of the Stockport Infirmary, has taken over the Victoria Nursing Home at Harrogate.—Last week the Mayor of Cardiff opened the new Nurses' Home which has been erected in Park Grove, in connection with the Cardiff branch of Queen Victoria's Jubilee Institute for Nurses.—The death at Hong Kong of Mrs. Gibbs, after a short illness, is announced. Mrs. Gibbs, who was known in the nursing world before her marriage as Miss Catherine McIntosh, was one of the six sisters sent to the Civil Hospital, Hong Kong, in September, 1890, at the request of Dr. Atkinson.

### Lectures to Nurses on Anatomy.

By W. Johnson-Smith, F.R.C.S., Principal Medical Officer, Seamens' Hospital, Greenwich.

### LECTURE II.—THE SPINE.

A glance at the spine will suffice to show that it is not a compact and continuous bony column as its common term would imply, but is made up of a long row of distinct bones. These constituent bones, the number of which varies in different subjects from 28 to 30, are called *vertebræ*, and so give to the spine the usual anatomical name of the *vertebral column.*

Twelve of these vertebræ, it will be seen, are distinguished from the rest in their giving attachment to the twelve pairs of ribs. These twelve bones are the *dorsal or thoracic vertebræ.*

Between the first and uppermost of these dorsal vertebræ and the lower surface of the skull there will be found a row of seven bones to which, from their position in the neck, the name of *cervical vertebræ* has been given.

Below the last dorsal vertebra with its pair of short ribs there are five distinct vertebræ, which form part of the posterior wall of the abdomen: these are called the *lumbar vertebræ.*

The last of the lumbar vertebræ rests on a relatively huge vertebra about as broad and long as the palm of the hand, which is wedged in between the two large and expanded bones at the lower part of the abdominal cavity. This bone, which forms the back part of what anatomists term the pelvic girdle, is the *sacrum.*

The terminal portion of the spine is made up of three, four, or, at the most, five very small misshapen segments called *coccygeal vertebræ*, which, in aged subjects, are often joined together to form a single bone called the *coccyx.*

The segments of the vertebral column, therefore, may be thus arranged from above downwards:—

7 cervical vertebræ

12 dorsal or thoracic vertebræ

5 lumbar vertebræ

1 sacrum

3 to 5 coccygeal vertebræ

we carry the eye along the spine it will be seen that the vertebræ show a general tendency to increase in size from above downwards, and that they also undergo a change of form. No two vertebræ are alike. Two contiguous bones may show a very decided resemblance, but if we compare two extreme bones of the column, as, for instance, one from the upper part of the neck with the fifth lumbar, very marked and puzzling variations will be observed. The vertebræ, however, are all built as it were according to the same plan, and their differences in form are due merely to variations of architectural detail.

A vertebra taken from the mid-dorsal region and surveyed from above or below presents in front a large and more or less rounded disc of bone, fig. 2, A, and, behind this, a continuous arch of bone, B, forming with the posterior surface of A a complete ring. Indeed, so far, the vertebra resembles a signet ring carrying a very large seal. Projecting from the arch, one on each side, are two lateral rods of bone, C, and from the centre of the arch behind springs a similar single outgrowth, D.

It is important to remember the names of these different parts: the disc of bone in front, A, is called the *body* of the vertebra; the ring behind this, B, the *neural ring*; the two lateral outstanding rods of bone, C, the *transverse processes*; and the single projection behind, D, the *spinous process.*

On taking a lateral view of the vertebra, fig. 3, we find some other structural details which should be carefully noted.

The narrow sides of the arch are attached to the thick body much nearer to its upper than its lower surface. Two notches, fig. 3, E E, are thus formed, the lower being much deeper and larger than the upper one. At the root of each transverse process will be seen a pair—one above, the

Fig. 2.—A Dorsal Vertebra (upper view).

A Body; B Neural ring; C C Transverse processes; D Spinous process.

other below—of out-growths of bone, each ending in a broad, almost vertical, surface, which in a fresh bone is covered by cartilage.

The notches are called the *intervertebral notches*, E E, and the out-growths of bone the *articular processes*, F F.

If we turn our attention from a single vertebra to the spine as a whole we shall find that the bones form a continuous and solid column increasing generally in width from

Fig. 3.—A Dorsal Vertebra (side view).

A Body; C Transverse process; D Spinous process; E E Intervertebral notches; F F Articular processes; H H Facets for heads or ribs.

above downwards in relation to the increased weight it has to support.[1] The neural rings form a continuous canal enclosing the spinal cord, which sends off nerves to all parts of the body through the orifices [*intervertebral foramina*] formed by the apposition of the intervertebral notches. The articular processes by their interlocking prevent undue lateral movement of the different vertebræ, and the lateral and spinous processes afford attachment to the ligaments which hold the vertebræ together, and to the muscles which move the different vertebræ and maintain the spine in the erect position.

[1] The lower part of the cerbical spine is a little broader than the upper part of the dorsal spine; the first four dorsal vertebræ forming a pyramid with its base upwards.

### A Nurse on the Royal National Pension Fund.

An article appearing in *Nursing Notes* of the current month by one of "The Second Thousand" of the Royal National Pension Fund strikes us as so illuminating and exhaustive that we reproduce it in The Hospital. It is as follows:—

As a nurse who has herself belonged for many years to the Royal National Pension Fund, I have been asked to write a practical note to nurses on the subject, both of belonging to the Fund and of making provision for old age. In the first place I should recommend any nurse who is thinking of joining the Fund, to call at the office and make her own inquiries from the Secretary. I did this myself, and, though I daresay after the interview the Secretary's opinion as to the business capacity of nurses had received another shock, I came away very grateful for the kind way in which my stupid questions had been patiently answered. But some nurses won't or can't do this, and that is why the editor of *Nursing Notes* has asked me to write "from a nurse to a nurse." I said when she asked me "that such a letter should be headed 'from a stupid to a stupid,'" but she thought that would not quite do.

How much can a Nurse Save?

The question of saving has to be regarded from two standpoints. Firstly, how much can a nurse save? and secondly, how is it best to invest such savings? How much a nurse can put aside during her working years to provide for old age depends on so many things. It is quite impossible to lay down any general rules about saving, and, therefore, for the purpose of this paper, I will take the case of a nurse of the ordinary age, at the ordinary wages, and with the ordinary health, who has no one dependent on her, or to whom she feels she ought to leave her savings, but who also has no one to help her either while she is working or when she is past work. I will take the case of such a nurse, and discuss how she had better provide for old age. In the first place, how much can she save? This depends, of course, on how much she is earning. I will conclude that she began to train at 24, that she is now 28, and has been thoroughly trained in general nursing and certificated for midwifery and massage. As she may have had to pay for these extra trainings, she will not have saved much before 28, but she starts thoroughly equipped for her profession. Of course I conclude that my nurse would endeavour to obtain a post at the highest pay possible, and to do this, she may perhaps have to somewhat sacrifice her inclinations to obtain a rather higher salary. If she takes a resident post in a hospital, as a district nurse, as a private nurse in an institution, her board, lodging and washing are provided, and these practically count for, at lowest, £50 a year on to whatever salary she receives, the two together making up what she would call her gross earnings. Her salary would be about £35, her gross earnings £85. The midwife, monthly nurse and the nurse masseuse working on their own account earn far more than these, and I think we may conclude that the thoroughly successful trained nurse earns on an average from £85 to £120. Striking a mean we will conclude that the nurse we are considering will earn £100 a year. It will be said that there are many nurses who must save who are not earning this. Very true, but if the nurse is thoroughly competent, which we conclude she is, and suitable in every way, she ought to be earning £100 a year, and if she is dependent on her own exertions for the future, she should certainly take steps to obtain employment that will bring in this. On the other hand, many nurses are earning a great deal more than £100 a year. We will conclude, therefore, that in her thirtieth year our nurse is earning this sum, we will hope that she will do so until she is fifty—that is, for twenty years. Now, how much ought our nurse to save? After making inquiries and consulting with nurses, who are actually saving this amount, and more, I have come to the conclusion that, other things being satisfactory, a nurse should save at least £20 a year. Let us say that the nurse will save £20 a year for 20 years; and to those who say it is too large a sum for them to put aside out of £100 a year, I can only assure them that I know of a considerable number who are saving £30 to £40 out of this sum. There are many who consider that the right proportion to save is half the salary, whatever that may be; in that case, those earning only £35 would not save so much as £20, unless, as is the case in district and hospital posts, about £5 worth of uniform is provided; this would make up the salary to £40.

Self-denial Needed.

I do not pretend to say that saving this proportion would not entail a considerable amount of self-denial, but if a nurse begins to put aside half her salary directly she ends her training, she will not feel it so much. It is what other working women have to do, and to do it without the compensations that fall to the lot of a nurse. Few other professions are so full of interest as ours—few have the drama of life so dramatically passing under their eyes. What other profession has the absorbing occupation of personally doing battle with the great enemy? And in those cases where the work may not be so interesting from the nursing point of view, though perhaps quite as really valuable, is there any other work where such kind friends are made? (how few nurses for instance need pay for their yearly holiday), or where those nurses, who have the true missionary spirit have a wider field of usefulness? Among other women who work for their living, nurses are regarded as most wasteful and extravagant; their childish expenditure on sweets, flowers, worthless present giving, having their photographs taken, theatre going, extravagant food on their days off, smart dress for holidays, etc., fills the ordinary worker with amazement mingled with contempt that women, belonging to a profession where the work itself is so full of interest in the present, cannot restrain themselves to save for that future that comes only too soon, especially to nurses; the age limit in our profession being quite as stringent and often as unjust as in other professions. We nurses know there is another side that could be presented to our critics, we need not deal with that; I only place before my profession what is said of us by others. Now, what had best be done with the £20 (we hope more) that is to be invested every year for twenty years? For a nurse, situated as I have described, I believe in a deferred annuity, *i.e.*, investing the £20 a year in some way so that at the age of 50 an annuity is paid for the rest of one's life. In this way a far higher rate of interest can be obtained than any nurse could possibly get by investing on her own account in any ordinary way.

What to do with £20 a Year.

Of course we all have some friends, very often good business men, who consider sinking money in any form of annuity, deferred or immediate, the most wasteful and unbusinesslike proceeding, and certainly if these advising friends could undertake to provide the nurse with a high, and at the same time safe investment for her savings, say at least 7 per cent., then the matter would certainly be worth consideration, and if the nurse has relatives whom she wishes to benefit on her death, she should not invest in an annuity. But we are considering the case of a nurse who has no relatives dependent on her, and she should be cautioned as to the safety of any investment for small savings that pays more than 3½ per cent. High interest for those who cannot personally watch the money market, always means risk—and the savings of working women are too precious to risk in speculation. I will take it for granted that this nurse wishes to invest in an annuity payable at 50. Nurses seem to find it difficult to grasp that at 50, or whenever the annuity begins, that they will have no longer any power over the principal. They forget that all the money paid for annuities has to be safely invested somehow, and that the reason the annuitants get such a high interest for their money when they come into their annuities is in consequence of a certain number of deaths of annuitants, that is sure to happen according to actuarial calculations, and this enables the survivors to enjoy the interest of the total money that has been paid in by those annuitants who die as well as those who live. Naturally, the annuities would be a good deal higher if the premiums were non-returnable and the annuity not due till 60. It is one of the greatest advantages of the Pension Fund that the money is not sunk until the age for commencing the pension is reached. But there may arise so many reasons for wishing to take money out before 50, that a non-returnable scheme can never be advised—and a nurse's hardest working and largest earning time is so often over by 50, that the later age to take out a pension cannot be recommended. As the office chosen is of great importance, some years ago, when the Royal National Pension Fund first started, I made many inquiries from business men as to its safety, and how its rates compared with other offices; I obtained quite satisfactory answers, which have certainly been justified during the last 12 years.

### The Question of Premiums.

I often hear nurses say that the premiums are so very high in the Pension Fund. I think if they will take the trouble to compare the Pension Fund with any other safe assurance companies, beginning with the Post Office deferred annuities, they will find that the rates compare favourably. There are also several other advantages connected with the Pension Fund that are not obtained in other companies where everything is on a strictly business footing and none of the personal difficulties or troubles of the policy-holders are taken into consideration. I have always felt that many of these objections are owing to the name of the fund, and would not arise if it was called the Royal Mutual Assurance Society for Nurses, or some name of that sort. We have all got preconceived ideas as to what a pension is. We feel it should be, in some way, a reward for long and meritorious service; and nurses feel it hard that when they have perhaps given the long and meritorious nursing service for 20 years, they have to pay for a pension as well. On reference to the tables I find that £20 a year for 20 years will bring a certain pension of £30 15s. 4d. Nurses should compare this rate with other companies and see if they give as much. I think they will find that there are very few offices that do, and on inquiry I have found there is some disadvantage, not at first apparent, if the rate seems higher than that of the Pension Fund.

### The Benefits.

Now what benefits do those who put into the Pension Fund obtain above this £30 odd, which, practically, they could obtain in several other offices? I think they obtain a great deal in the Pension Fund. For instance, in some cases, especially if the nurse holds a first thousand policy, the annuity would amount at 50 to about £40. Now the sum put aside by the nurse during 20 years, without interest, is £400, with compound interest at 3½ per cent. would amount to £565 11s. 10d., therefore the nurse at 50 would be receiving over 7 per cent. for her savings. I do not think that anyone could invest in a better way than this. There are other reasons which should induce nurses to endeavour to invest in the Royal National Pension Fund. One is the great kindness which nurses meet with if they are unable, from some reason or other, to pay up their premium regularly—they can borrow the money to do so, on paying a small interest. If it is quite impossible for them to make up their payments, the excellent Junius S. Morgan Benevolent Fund comes to their help, and in cases of hardship where nurses have really made a brave struggle to subscribe to the fund, for a considerable time, and in the end have failed to make provision for their old age, or to keep up their payments, I know instances where this Benevolent Fund has been most generous with its help. Nurses, at many times, have been given a much needed rest and holiday, and others have been helped to make up their payments. In some cases where they have come into their pension, which has been too small to enable them to get along, the Benevolent Fund has supplemented this with another pension. Another advantage is, should this nurse, whose case we have been considering, wish to withdraw from the fund any time before the age of fifty, she can do so by giving one month's notice at any time, except during the first two years. This is a most excellent regulation, because it prevents nurses drawing out directly after they have begun to put in. By the end of the first two years they have got more accustomed to saving, and would not draw out their savings for any trifling reason. Two and a half per cent. compound interest is allowed upon all savings withdrawn after these two clear years. There is a charge of five per cent. for the cost of administration on the gross monthly premiums received. On this matter of withdrawals the Pension Fund comes out better than other offices with which it has been compared.

### Hospital Nurses.

Nurses who prefer hospital work (and therefore, as we know, will not receive nearly such high salaries as those who are in private work) should see that they enter for training at a hospital that is affiliated to the Royal National Pension Fund. Most of the recognised training schools are, and this in a great way compensates the nurse for her smaller salary. If she joins at once on being appointed to some hospital post, say at thirty years of age for the pension at fifty of £22 10s. or £30, she will have to pay half, the hospital will pay the other half, so she is earning practically this much more salary. Say she leaves the hospital for other work after five years, that money is hers, and she is well started on a thrifty path, and the better pay that she may have left the hospital to obtain will enable her to keep up her payments. I can hear nurses saying, "So I am to scrape and scrimp all the time life is most enjoyable, to get what? A sum a year at 50 on which I cannot live in any comfort, I had better go to the workhouse when that time comes and enjoy life while I am young. Yes, my sister nurses, you say this at 30. But will you say the same as you near 50, with nothing saved, nothing certain (however small) coming in, that would enable you to take the lighter, but less well-paid work, that would very likely enable you to work till 70, and for another 20 years still save a considerable sum on which with your pension you could live in comfort for the rest of your days. How many nurses would be able to start with renewed vigour if at 50 they could take a comfortable six months' holiday without feeling it unwise to let a single case pass by. If the worst comes to the worst, and you cannot work at 50, or have to take a long rest, it is much easier to receive kindness and hospitality if there is a certain small sum coming in for pocket money, and the extras that illness entails, and for which it is an added bitterness to have to beg. The offer of a long rest in some country home can be accepted, but it is preferable to be able to pay washing and travelling expenses. I have several friends who are within a year or two of 50, when they get the pension for which they have striven very hard, for the women of 50 now were 38 when the Pension Fund began, and the premiums they have had to pay are far higher than those I have been considering. One of these looks forward at 50 to the possibility of a long rest; another to lighter, another to more congenial work; another to the ambition of her life—a little garret (as we say in the North, "a bein' of my very own for my bits of sticks"). They all say the struggle has been worth while, and that they are sure they would never have saved any sufficient amount if it had not been for the constant drive of the quarterly payments to be kept up. The majority also say that these payments would never have been kept up without the friendly hand, help and encouragement of the Pension Fund itself.

An Appeal to the Unbusinesslike.

I have only one more thing to say and I have done, and that is to entreat my fellow nurses to make their own inquiries from headquarters. Of all the working women that I have come across we nurses are the most unbusiness-like. We are wildly credulous, or comically suspicious, there seems to be no half-way house. We will believe anything told us by anyone, without in the least considering if our informant knows what she or he is talking about; I fear we again repeat what we have heard, and most wonderful are the particulars as to the Pension Fund that have come to my notice. This would not matter if nurses took the trouble to verify these reports; this they won't do, and are only confirmed in their own inclination not to begin saving at all—a persuasion which needed no encouragement. It is just as absurd for nurses to come to such definite conclusions about investing money, without taking reasonable advice, as it would be for business men to come to definite conclusions as to the management of a ward, or other nursing details, without consulting the experts in the subjects. Those who wish to know more about the Pension Fund will find a most kind and patient expert in the Secretary; the direction is 28 Finsbury Pavement, E.C. In the words of the immortal Captain Cuttle, "when found make a note of."

### The Krankenhaus in Hamburg.

A Visit by an English Nurse.

Visiting Hamburg some weeks ago, I was advised by my friends to go and see the new "Krankenhaus," situated in one of the suburbs of the town. It is considered to be one of the finest hospitals in Germany. Founded some 12 years ago, built of solid red stone with 2,000 beds ever open and ready for the suffering public, it has a most imposing appearance. It is comprised of many separate houses, which, tastefully arranged about a beautifully laid out piece of land, remind one singularly of a well built village, taking over an hour to view satisfactorily. These houses have huge numbers on them, and are arranged according to the four different classes, all patients having to pay proportionately. The first class, who with the second class enjoy separate rooms for each separate member, pay 10 marks and 6 marks a day respectively; the third and fourth class, who are in wards with 30 or 40 beds, pay respectively 4 marks and 2 marks a day; the last named wearing a costume of white linen and with their white caps look singularly like cooks when they are promenading in the grounds. The very poor are provided for from the general hospital fund, but as it is made compulsory for them to place themselves when they are well and at work under the protection of certain associations and clubs, it is very seldom that they are unable to pay independently. Many rich people have "Freibetten" (free beds), to which are admitted, free of charge, poor deserving cases, personally known to them.

The Operation Theatres.

Besides the small operation rooms which adjoin some of the largest wards, an entire house is kept only for operation theatres. Each "operation's saal" has its own anæsthetic chamber adjoining it, in which hang entire suits of antiseptic clothing—white linen and guttapercha—marked with the names of the various operators and their assistants. Different kinds of operations are carried on in particular theatres, and there are theatres for men and women respectively. They are exceedingly well lighted by huge bay windows which almost measure from the floor to the ceiling. The walls and floors are of polished marble, and the lotion stands, washstands, and instrument tables of spotlessly white "marmorite." The operating tables appear to be of enamelled metal, and have the usual heating apparatus, and are padded with white mackintosh mattresses. Over the washhand stands are large sand glasses, which, set to run through, specify the time considered necessary for the washing and scrubbing of hands; and there are foot pedals instead of taps for turning on the water. Adjoining the biggest theatres are rooms set aside for sterilising purposes; dressings and towels are rendered aseptic here, in dry heat, and boiled in carbolic lotion. Great boilers, with businesslike looking men attired in white linen tending them, fizzle and hiss, and there are white enamelled metal receptacles containing piles of soiled towels which will presently be thrown into boiling carbolic lotion.

Twelve to Fifteen Cases Daily.

It is the rule that all operations must be performed in the hospital to which the particular doctor of rich or poor belongs. No doctor will perform an operation in a patient's own home, so that there is always extra work to be done in the theatres, and as the usual amount of operations daily is from 12 to 15 cases, the theatre sisters, clad from head to foot in white linen overalls, are kept very busy. The whole antiseptic system is carried out admirably, as is, indeed, the case in even the smallest hospitals in Germany. There, especially, the work in laundry and kitchen section is carried on like clockwork, likewise the business part of this great hospital. It is headed by a most imposing-looking "Direktor," and countless busy-looking secretaries.

Disorder in the Wards.

But in direct contrast to this picture of cleanliness and order are the large well-built wards, in which one misses to a lamentable degree the necessary discipline. The probationers chat and laugh intimately with the sisters, who look anything but neat, and walk easily and comfortably about the wards apparently oblivious of the fact that the floors are somewhat belittered with bits of wool and crumbs, emptied mugs waiting to be removed, dressings by the bedside, and very often spilt milk and other fluid left to dry on the stone floor. The beds are ugly, there are no flowers, the air is stuffy, and in some wards the beds appear to be too close to one another.

A Hint to the Authorities.

In spite of the nurses being apparently permitted to smack the naughty children, they seem to be very kind. A good many of them are ladies, though a large majority of them are of the lower classes. They are not overworked, the recreation is good, and the period of training is two years. If the German hospital authorities would realise how necessary discipline is for the satisfactory management of a large staff of nurses, and would put sisters accustomed to the training of probationers at the head of the wards, there would soon be a marked difference. It is disappointing to see a hospital, perfect in every other respect, fall short of perfection from the lack of order and discipline in one of its most essential branches.

### Nursing in Workhouse Infirmaries.

### THE REPORTS OF THE INSPECTORS.

The annual report of the Local Government Board for 1900-1901, which was issued this week, contains some interesting references to the nursing in workhouse infirmaries.

Mr. J. S. Davy, inspector for the district comprising the counties of Sussex, Kent, and "part of Surrey," reports:

The general nursing continues to improve, and, except in one case, there has been no obvious friction in the working of the nursing arrangements. A scheme for utilising the services of the workhouse nurses for the nursing of the out-door poor which was adopted by the Chichester Guardians about five years ago, presents several points of interest, and may in some unions suggest a solution of the difficulty of maintaining a staff of nurses which shall be sufficient both in numbers and in qualifications. The guardians appointed an additional nurse at the workhouse, raising the total number employed to four, and assigned to them the duty of visiting and attending sick cases in receipt of out-door relief. Each nurse is told off in turn to undertake the duty for a week, nursing orders being only given to persons who are actually receiving relief. The nurse in charge has to visit each case as often as may be required and to report upon it to the superintendent nurse. Where necessary she has to administer certain medicines, as, for instance, stimulants. The guardians are completely satisfied with the results, as they are of opinion that cases of sickness are more rapidly cured than if they were under the old system, and that the old and chronic cases are much better treated. The visits of the nurse in her official dress cannot be concealed from the neighbours of the pauper, and this is held to be a deterrent to many applications from persons who are able to pay for themselves. Certainly the change has been concurrent with a large diminution in the number of paupers relieved in the Chichester Union. The net amount paid in outdoor relief has dropped from £1,359 in 1897 to £921 in 1900, while the number in receipt of relief has decreased by about 30 per cent. In considering the question of the possibility of the adoption of a similar scheme elsewhere, it must be remembered that the Chichester Union covers a very small area, being only 1,595 acres.

Mr. J. W. Preston, inspector of the districts comprising the Union counties of Bedford, Hertford, Huntingdon, Middlesex, Northampton, and Cambridge (Wisbech excepted), reports:

No doubt the difficulty in providing trained nurses for our workhouse infirmaries, especially those in rural districts, is very considerable, but I gladly record that guardians are gradually doing their best to provide not only adequate nursing for their sick, but better accommodation for the nurses, which is of as much consequence as the salary itself. With that object homes are being, or are about to be, erected at several of my workhouses. Unfortunately, the necessity of night nursing is a question which many guardians consider of little moment, as they regard the services of an inmate as sufficient, but that system, I am glad to say, is gradually though slowly being abolished. I should be very pleased to see paid labour for scrubbing, etc., the rule, and not the exception, in our sick wards.

Mr. Baldwyn Fleming, inspector for the district comprising the Union counties of Dorset and Southampton and part of Wilts and Surrey, reports:

Workhouse nursing has made some advance during the year, and the Order issued to Basingstoke and Farnham has an important bearing in this direction. The effect will be watched with interest, and if no administrative inconvenience result, it may be deemed safe to extend its provisions as regards the special care of the sick wards to all unions where there is a superintendent nurse. This would remove one source of possible friction between the nurses and other officers, and would relieve the master and matron of a duty which, by the regulations, they are called upon to perform, but which they often leave aside because their common sense tells them not to disturb the sick wards after the patients are settled off for the night.

Guardians have continued to allege difficulty in obtaining competent nurses for rural workhouses, and assume that this is caused by a lack of persons qualified to apply for the posts. It, however, appears to be very doubtful whether this is the true ground of difficulty, and whether it is not rather because rural guardians do not make the posts sufficiently desirable to attract good candidates.

Wards, duties, facilities for efficient nursing, sanitary appliances, apartments, assistance, attendance, rations, cooking, service, companionship, recreation, unbroken monotony of work under trying conditions, discharge of workhouse medical officer's duties; all these and many other items must be adequately met before the difficulty of obtaining good nurses may fairly be assigned to lack of candidates instead of to bad conditions of service.

Nursing questions occupy a foremost place among those with which lady guardians can deal, and upon which their advice and help have been of the greatest value since the Board's Order upon nursing in workhouse sick wards was issued.

Many of the suggestions which are put forward by those who are not acquainted with the administration of workhouse establishments for improving the supply of nurses are evidently impracticable. For instance, that the Board should train nurses, when the Board have neither hospital or patients, nor funds to provide them, if their provision were practicable, which it would not be.

Again, it has been said the probationers trained in Poor Law infirmaries should be bound to work for Poor Law institutions. Such a pledge would at once deter instead of attracting candidates.

The demand for nurses for all purposes has increased so rapidly that guardians cannot expect to obtain them as easily as if there were less competition for their services. There is all the more reason why guardians should set about removing the distasteful incidents which occur in their own establishments. They may feel assured that if they make their positions pleasant, the nurses will take them. The workhouse medical officers are not always without reproach in the matter. Entries recently taken from porter's books in several unions show that some medical officers frequently only stay for a few minutes in the house, and very rarely long enough to make an examination of the bodily condition of the patients and of their surroundings. The medical officer should certainly, and with sufficient frequency to ensure the object, thoroughly examine each of the patients, and particularly the bed-ridden ones, as to bodily condition and cleanliness, also the beds, bedding, appliances, and all other details connected with the proper treatment and nursing of the cases. Unless the nurse see that the medical officer is himself thoroughly interested in his patients it is not to be expected that she will long continue to be so. A good medical officer will keep a nurse up to the mark, and (although to a less degree) a good nurse will keep a medical officer up to the mark, but when the nurse finds that the medical officer is careless and performs his duties in a perfunctory manner, she loses heart and comes to lack the care and pride in her work which, under better circumstanes, care a most satisfying feature of her daily life, and which, when absent, leave a void to be filled by discontent.

### Portsmouth Workhouse Infirmary.

Mr. Fleming has a special note in reference to the training of nurses and probationers in the Portsmouth Workhouse Infirmary. He says:

The training of nurses and probationers in the Portsmouth Workhouse Infirmary has been tested during the past year by an examination held by Dr. Bryant, Assistant Physician to Guy's Hospital.

He made this report: "I have tested the knowledge of the nurses of the Portsmouth Union Infirmary by means of a written and an oral examination. I have much pleasure in stating that they have done remarkably well. Of the eight first-year nurses who presented themselves, six qualified; one, viz., Nurse Ada Cottrell, with distinction. Two did not obtain the necessary number of marks, and these I rejected.

"Of the 16 second-year nurses, 14 qualified; five with distinction, viz., Nurses K. E. Gaul, E. M. Payne, E. Gyles, E. M. Worthington, and L. Hanham. Of the two rejected I recommend that Nurse B. C. Price be granted a first year's certificate.

"Those who qualified showed a sound and practical knowledge of their work, and reflect the greatest credit on their teachers. It is evident to me that their lecturer has taken a great interest in them, and has devoted a good deal of time in bringing them to their present state of excellency.

"They also showed a most intelligent interest in their work and I feel sure that this scientific teaching they are now having will not only increase the efficiency of the nursing, but will enable the Portsmouth Union Infirmary to turn out nurses equal to any in the United Kingdom. I congratulate the board on their farsightedness and liberality in commencing, in their infirmary, the scientific and efficient training of their nurses."

This is the first occasion upon which an independent test of the quality of the training has been applied, and the result would seem to be remarkably good.

Mr. R. I. Dansey, inspector for the district comprising the Union counties of Salop and Chester, and parts of Hereford, Stafford, Worcester, and Derby, reports:

The arrangements for the nursing of the indoor sick are improving throughout the district, and the number of nurses has been increased, so that, taking an average throughout the workhouses, there is now a nurse to about every 18 beds.

The difficulty principally lies in the small workhouses, where the number of sick is often quite insufficient to employ a nurse's whole time. In three workhouses of this character, where there are seldom more than three or four cases—mostly senile decay or bad legs—confined to bed, the guardians have had to entrust the matron with the supervision of the nursing, allowing a person, appointed as assistant to the matron, to help her in the work, while the master is instructed to engage the services of a trained nurse on any emergency.

This arrangement is, of course, open to objection, and perhaps equally so is the suggestion of allowing a nurse in such a workhouse to be placed under the control of the medical officer when he is in charge of the workhouse and adjoining district—to be employed among the outdoor, as well as the indoor, sick at his discretion.

Mr. H. Jenner-Fust, inspector for the district comprising the Union counties of Lancaster and Westmorland, and parts of Cumberland and the West Riding of Yorkshire, reports:

The "Annual Nursing Return" for the district is of a satisfactory character. Although, comparing January 1st, 1901, with January 1st, 1900, there has been an increase of 259 in the number of inmates under the care of the medical officers, exclusive of imbeciles and epileptics attended in special wards, there has been a corresponding increase of 23 in the number of nurses employed, including superintendent nurses, assistant superintendents, charge nurses, assistant nurses, and probationers. The numbers for January 1st, 1901, were 9,623 sick and 755 nurses, which show a decrease, though a very slight one, in the number of patients per nurse.

During the six years since the return was first issued the sick have increased in numbers by 793, or 8·9 per cent., while the nurses have increased by 250, or 49·5 per cent., and the number of patients per nurse has diminished from 17·48 to 12·74.

The main points with relation to the sick may be tabulated thus:—

| Date. | Number of Sick. | Nurses on Day Duty. | Nurses on Night Duty. | Total. | Increase. | Number of Patients to each Nurse. | Number of Pauper Attendants. |
|---|---|---|---|---|---|---|---|
| December 1—1894 .. | 8,830 | 396 | 109 | 505 | 40 | 17·48 | 279 |
| January 1—1896 .. | 9,228 | 433 | 128 | 561 | 56 | 16·44 | 260 |
| 1897 .. | 9,029 | 458 | 125 | 583 | 22 | 15·48 | 250 |
| 1898 .. | 9 182 | 478 | 141 | 619 | 36 | 14·83 | 286 |
| 1899 .. | 9,084 | 510 | 154 | 664 | 45 | 13 68 | 253 |
| 1900 .. | 9,364 | 565 | 167 | 732 | 68 | 12·79 | 206 |
| 1901 .. | 9,623 | 579 | 176 | 755 | 23 | 12·74 | 191 |

### A Cyclone on the Veldt.

By an Army Reserve Sister.

The readers of the following description of a cyclone will kindly bear with the writer and draw upon their own imaginations in order to give colour to the episode which occurred on the veldt, about three miles from Kimberley. I must plead youth and inexperience in the flowery paths of literature, and though my account may be neither graphic nor picturesque, I will do my best to describe truthfully what happened.

It was the latter end of March, 1901, and about 5.30 in the evening of a day which had been scorchingly hot. The sunset was peculiarly beautiful, one of those gorgeous sunsets for which Kimberley is noted, and as a matter of fact almost the one redeeming feature in that sand-bound town of the veldt. I remember distinctly having come outside my hut to admire the sky, which had changed from the palest tints of pink and violet to a deep blood-red, the whole atmosphere being imbued with the strange lurid light. Suddenly in the distance was heard a low, angry rumbling, getting louder and louder. "Boer guns!" whispered one. "No, mine blasting," said another. I may mention that the camp was located over a diamond field. The noise came nearer, and sounded now more like mighty waves breaking on a rocky shore. Then an old campaigner shouted, "Get into the hut and hold on, it's a cyclone." In we rushed and fastened the door, and not a moment too soon; through the windows we could see it coming—an enormous, apparently solid looking wall of sand which swept everything before it; bell tents and marquees went down like skittles, and corrugated iron roofs were torn off in the fraction of a second. Then it was our turn; a gasp for breath, a mighty rocking of the whole hut, and we all turned to see what was left of us. The cyclone had broken over us and apparently done no mischief beyond the whole row of windows broken and a few bottles smashed. It, however, had not finished its work, and gathering strength as it went, broke again over the medical section of the hospital; nearly every marquee went down, and finally we could see it rolling away in the distance far beyond us. When it was over, we could turn and witness the havoc it had wrought in a fifth of the time it has taken to write this. The state of the poor patients was pitiable, they could be seen on every side crawling from underneath the debris—bedsteads, tables, chairs, and in fact all the equipment of the hospital, was lying about in hopeless confusion, and utterly beyond repair—basket chairs were torn to ribbons and iron beds bent like thin pieces of tin into all shapes. The bedridden men fared worst, having to remain until they could be extricated from the muddle. Fatigue parties were quickly organised, and the patients conveyed to any available shelter. A few huts which were already opened were filled to overflowing, and the rest of the men were put into the linen stores, cook-houses, and wash-houses; in fact, anywhere under a roof for the night. Next morning, early, the work of repitching the tents commenced, and by afternoon most of the patients had been transferred to their marquees.

To our horror, at 6 o'clock the same evening, the whole thing happened again; the last state being worse than the first, as a violent storm was raging at the same time, and more tents collapsed than on the previous evening. When we had somewhat recovered from the shock we saw that only seven tents out of the hundred or more comprising No. 11 General Hospital were left standing, and they were in a hopeless state of rags and tatters; the rest were level with the ground. Fortunately, and no one could understand why, there were very few casualties, and those only cut heads and a few slight injuries to limbs.

Next morning the General rode round the camp, or what remained of it, and ordered huts to be put up in place of the marquees, which was speedily done. I may add that bedding, articles of clothing and lighter things, were being brought in daily for weeks after, having been blown and carried by the cyclones for miles across the veldt. It was roughly estimated that over £2,000 worth of damage had been done in the few minutes of the two cyclones. I never wish for such another experience.

### Everybody's Opinion.

[Correspondence on all subjects is invited, but we cannot in any way be responsible for the opinions expressed by our correspondents. No communication can be entertained if the name and address of the correspondent is not given, as a guarantee of good faith but not necessarily for publication, or unless one side of the paper only is written on.]

### NURSES' READING SOCIETY.

"Miss Moberly" writes: Owing to an unfortunate omission Miss Milligan's name was not inserted in the list of prize winners for the year just past. She has gained a prize of 7s. 6d. and her name should have been inserted between those of Miss Till and Miss Lockwood. May I at the same time make known to those interested in the reading society that my address, instead of being 24 Portland Place, is now Belsize Cottage, Belsize Lane, N.W. It is greatly hoped that new members may join the society and enable it to continue its existence now seriously threatened.

### ON DUTY AFTER HOURS.

"Justice" writes: A short time ago I read a letter in your columns from a "Tired Night Nurse," who complained of being kept on duty an hour or so after the appointed time. I feel much sympathy with her. Two night nurses, with whom I am well acquainted, are kept on duty three-quarters of an hour after the appointed time every morning. They are left in charge of 100 patients every night, many of whom need a great deal of attention, and it is impossible to finish their work until three-quarters of an hour after they ought to be off duty. When at last they are finished they are completely exhausted. I might add, too, that one of these night nurses is a probationer of three months only. It seems to me that if a nurse gives of her best to her work, a little consideration is due to her. Then, one word more upon another subject. What are lunatic asylums for? On an average 20 out of the 100 patients in the hospital I allude to are real lunatics. Other patients who are very ill are constantly kept awake by their shouting, etc. In fact one man remarked: "Why, nurse, talk about a hospital, it is more like a luny ward." Surely when a girl applies for a general training, it is unfair to inflict lunatics upon her.

### THE SCARCITY OF NURSES IN WORKHOUSE INFIRMARIES.

"An Infirmary Nurse" writes: I, too, have taken keen interest in your remarks about "The Scarcity of Nurses at Workhouse Infirmaries," but I cannot help feeling that "A Poor Law Nurse" is just a little too scathing in her remarks about Poor Law Nurses. Perhaps I have been exceptionally lucky, but during the four years I spent in a workhouse infirmary I never met a nurse, or sister either, who, if they were not ladies by birth, most certainly were as far as feelings, education, and general behaviour were concerned. On no account could you have applied one of "Poor Law Nurse's" horrible adjectives to them. The matron, doctors, and sisters worked hard to train the probationers thoroughly, and in the majority of cases succeeded in their efforts. As to it depending on the nurse's own efforts whether she gets trained or not, I think if a nurse does not make some sort of effort to try and learn, at any rate to remember what she is taught, it is rather a waste of time to contemplate training her. I admit that some of the smaller infirmaries which are not training schools do have an inferior class of nurse, but I think if "Poor Law Nurse" were to take the trouble to find out, she would ascertain that the large majority of workhouse infirmaries are training schools, also that they have ladies as matrons, and well educated, refined women as sisters and nurses. People who are ignorant of the real state of affairs are only too ready to sneer and look down on infirmary nurses, and I, coming under that category myself, must protest most strongly against being spoken of as "fast, flighty, drunken, lazy, ignorant and underbred."

"Lady Superintendent" writes: As lady superintendent of a workhouse hospital containing over 600 beds, may I attempt to give my opinion on the letter of "A Poor Law Nurse" in The Hospital of October 5th? She says, "As a rule the Poor Law nurse has no pretension to being a lady, or even to being ladylike. Fast, flighty, drunken, lazy, ignorant, and underbred, are adjectives which may be applied to the present Poor Law nurse, with only a few exceptions, in most of the infirmaries." I beg to say I have a large number of nurses and probationers. They are bright, educated, quiet, sober, and modest, ladylike women, drawn from a good social grade. They are the daughters of clergymen, doctors, and other professional men. I have not had one drunken nurse on my staff since entering upon my present post several years ago. As to being ignorant, they certainly are not. The training and work are good. The sisters or charge nurses have been trained in London and good provincial general hospitals, besides which I also promote probationers of my own training. They take a great interest in instructing those under them. My nurses have hard work, and they do it, having the patients' welfare at heart. Probationers receive three years' training with medical, surgical, and midwifery lectures twice weekly during the entire course. Examinations are held at stated times; at the end of the third year a final examination is held and certificates granted to those who are successful. About 30 nurses have obtained L.O.S. certificates and we are still sending candidates up. Probationers, after leaving, have obtained appointments as charge nurses and sisters in general hospitals, also under the M.A.B., and a probationer of my training is a superintendent in one of the field hospitals at the seat of war. I myself formerly was a 'charge nurse' in a workhouse infirmary and was fortunate enough to meet nurses similar to those I have on my staff. I should very much like to know where "A Poor Law Nurse" obtained her unique experience, and only hope that in her future appointments she may be more fortunate.

"Hospital Sister" writes: In reply to "A Poor Law Nurse," who suggested a few reasons for the "scarcity of nurses at the workhouse infirmaries," I should say that she has been most unfortunate in her training and experience and she certainly knows little of hospital work. I have had experience as sister both in hospital and infirmary and although I have always found the hospital nurse far superior to the infirmary nurse, I certainly have never come across nurses such as your correspondent describes. I think it depends not altogether "on the nurses' own efforts whether they get trained or not" so much as on those over them. "A Poor Law Nurse" is quite right in saying that "the training is poor and that in many cases the sisters are unable to teach the nurses," but she is very wide of the mark when she says that "there is no reason why the training should not be the best in the world, because in the absence of students the nurses get far more real nursing than in the general hospitals. In the latter, too, the sisters do a great deal of the nursing which in infirmaries is given to nurses." The training in infirmaries can never be so good as in general hospitals, for the simple reason that in hospital every case, more or less, is acute—in infirmaries acute cases are the exception—which, with the varied and excellent staff of a medical school, keeps the hospital nurse from falling into a groove inevitable where, as in infirmaries, chronic cases are the rule and where one or two doctors only go round. Then, the infirmary nurse has nothing in the way of clinical lectures to benefit by, which but for the presence of students would not exist. Also the sisters in hospital being hospital trained, are able to and do teach the nurses, but as for them doing the nursing left to nurses in infirmaries, I hardly think that is the case. A hospital sister supervises, and in helping with bad cases, teaches the nurses under her, but does not do the actual nursing. If infirmary sisters knew a little more and took more interest in their work, they would be better able to supervise and teach the nurses which, of course, would raise the standard of infirmary training. No good sister leaves the nursing entirely to the nurses, although I have seen it done in an infirmary. In that case the sister, instead of having shown the nurse what to do, simply stormed at her when something went wrong, a method of management not likely either to improve the knowledge of the nurse or the "tone" of the ward. Were "A Poor Law Nurse" to spend a week in a hospital ward, I do not think she would need longer to convince her of the large amount of real nursing that falls to the lot of the hospital nurse. Even with students (who are students and do not do the nursing) the hospital nurse has certainly far more real nursing than the infirmary nurse can have, and I fancy that "A Poor Law Nurse" would feel a little "out of it" were she suddenly planted in a hospital ward to do the little real nursing she fancies there is to be done. I have the L.O.S., and found no difficulty, though trained in a general hospital, in obtaining it. But, certainly, the L.O.S. "does not make a nurse."

### NURSING IN WORKHOUSE INFIRMARIES.

"Observer" writes: The subject of nursing in workhouse sick wards and infirmaries is very much to the front, and it is important to look facts in the face. Theories are useful, and without them we cannot expect practical results; but theories will avail nothing until the existing defects in the system of Poor Law nursing are grasped, not by a few interested workers who in their wisdom see how deplorable matters are, but by the country as a whole. The guardians of the poor must begin to realise their responsibilities, for it seems that the Local Government Board are unwilling to interfere with the decisions of boards of guardians, even when their own Orders are evaded, and the recommendations of their own inspectors totally ignored. A review of various reports issued during the last few months on matters connected with the administration of country workhouse infirmaries points to a laxity of system truly regrettable. The state of things at Walsall Workhouse Infirmary has been recently commented upon in The Hospital. It will be remembered that the Medical Officer had informed the Guardians that he had at night "been compelled to call up a nurse who had been on duty all day." He stated that ten men were dangerously ill and required "frequent and indeed constant attention." The priest who had been summoned to a (presumably dying) patient in the night, also felt it his duty to draw the clerk's attention to the fact that he found a probationer in charge of two wards containing sixty patients, in one of which three men were delirious. This state of things existed in spite of the fact, as stated by one of the guardians, that Mr. Wethered, the Local Government Board Inspector, had some time ago made certain recommendations which had evidently been ignored. This same guardian remarked that if those recommendations had been adopted the Board would not have been listening to those serious statements Does it seem right that the Local Government Board should be so unable to enforce their inspectors' recommendations? Does it not strike us as futile that the inspectors' work should bring such a poor result? Again we heard not very long ago of the medical officer at Dereham reporting that the duties of the nurse had grown so heavy that he felt compelled to ask the Board to appoint an assistant to relieve her. One of the guardians inquired if the woman who had been acting as cook might not be capable of fulfilling the duties. The question was referred to the master (not the doctor!), who said that he considered "the woman could quite well undertake the position of assistant to a nurse," and that if she would be willing to accept it he "would be ready to give her a trial." This, in spite of the doctor having reported that "the workhouse was fast becoming an infirmary, and that there were cases requiring constant attention," and that it was his opinion that one nurse could not do both day and night duty. It is stated that the matter was left in the hands of the master! We conclude that the cook undertook the night duty! In the report of a meeting of the Boston Board of Guardians headed "More Work for the Master," we read of a discussion on the organisation of the infirmary. One of the nurses had resigned, and it was not hidden that the purport of this discussion was to try and evade the letter of the law in reference to the appointment of a superintendent nurse. The master had told the Visiting Committee that if, instead of having three nurses or two nurses and a probationer they would appoint two nurses and a night attendant, they could so avoid having a superintendent nurse! This proposition was accepted by the Guardians, and it was decided to point out to the Local Government Board that the number of patients in the infirmary had recently decreased and to ask their sanction to the appointment of a head nurse, an assistant nurse, and a night attendant. The cases referred to in this brief sketch illustrate two distinct points in the administration of rural workhouse infirmaries: 1. Laxity of system; whereby the recommendation of the Local Government Board Inspectors may be totally ignored and by which, for example, a cook may be appointed as assistant nurse at the recommendation of the workhouse master and a probationer may be left in charge of 60 patients at night. 2. License of Guardians, who can so evade the instructions of the Local Government Board as to be able to arrange at the suggestion of the workhouse master that a superintendent nurse should not be appointed because they feared friction between herself and the master. These points exemplify the truth of a recent remark made in connection with the organisation of workhouse infirmaries—that no real advance will be made until "one uniform set of conditions, one uniform set of rules" prevail.

### QUEEN ALEXANDRA'S IMPERIAL MILITARY NURSING SERVICE.

"S. S." writes: I notice your note in "The Hospital" of October 5th, inviting nursing notes, criticisms, etc. May I be allowed to pass some remarks with regard to the regulations for the new Army Nursing Service, so fully and admirably retailed in your paper? I may have some claim to an opinion on the subject, having the practical experience of two years' work in the service, both in station hospitals at home and in the field in Africa, following years of experience as sister in a civilian hospital. First let me testify to the excellent work that has been done, both at home and abroad, by the R.A.M.C. officers, and of the service sisters, though, of course, few institutions—even military ones—are so perfect but that they can be improved. For instance, while right system and method make for the comfort of all concerned, sick or well, excessive red-tapeism does not. Neither because precedent is a sound principle to go upon need we be hide-bound with custom. The future "Queen Alexandra Imperial Military Nursing Service" will, we trust, yield the correct blend of systematic military preciseness with the wider and more up-to-date civilian methods. The first improvement I recognise is that the sisters are to be virtually the heads of their wards (as in civilian hospitals), not the mere figure-heads they are at present. The nursing will never be satisfactorily done until the responsibility of the nursing rests on trained women nurses, and not on male orderlies. For the sick-room is not a healthy man's sphere, while nursing is avowedly one of the vocations for which woman is most fit, and a gentlewoman's sympathetic tact and delicacy of thought and touch are gratefully felt by even the roughest patient. And men cannot be expected to do for a mere living, as women will for the love of the work. Now that sisters are to be accorded their right position in the work of the wards, it is time that they should also receive the respect due to that position. It is rather galling, under the present *régime*, to find that the whole ward "stands to attention" on the occasion of the visits of the sergeant-major, while few of the orderlies trouble to rise in the sister's presence; they will even take her orders sitting on the beds, and often do not remove their pipe from their mouths (the latter applies to field service, where smoking is, of course, allowed in the tents). Indeed, the orderlies are within their rights, according to the old R.A.M.C. regulations, to refuse to take orders from the sisters at all, the ward-master being their nominal head. It seems to me that not only should the sister have control of the sick ward, and the nurses therein, whether male or female, but that they should be entitled to salute from non-commissioned officers and men. This mark of respect is accorded to all other officers, combatant and non-combatant, and why should it be denied to the women because of their sex who hold the rank of officer, even if they were not saluted from courteous motives? I think this is a matter which should be noted in the Regulations. To hark back to the new Nursing Service, while it is of the utmost importance to enlist gentlewomen only in this branch (the nurses eventually come to the rank of sister), and to have well-trained nurses always, it is expecting too much to think that any nurse of three years' thorough training would enrol herself for another three years' probation, holding a lower position during the second three years than she occupied at the end of the first three, when staff-nurse in her civilian ward. And, unless the orderlies hold a purely subordinate place in future, as scrubbers, lavatory attendants, etc., there is really not scope in the military wards for sisters, nurses, and nursing orderlies. It is much to be desired that the nursing authority and duties should be vested entirely in the sisters and nurses; yet some trained and experienced nurse-orderlies will always be required for field-service. I am putting forward the difficulties of the proposed scheme, hoping that someone may suggest the necessary amendment. Finally, we all wish the work God-speed, and trust that the future Army Nursing Service may in no way fall below the ideals of the first Army Nursing Sister, Florence Nightingale; and that our military hospitals will be as comfortable for the patients, and stand as high in medical and surgical efficiency as the foremost of civilian hospitals. This is surely not an impossibly Utopian idea, for our State institutions should be second to none.

### Examination of Nurses at the Royal United Hospital, Bath.

The final examination for third-year nurses was held at the Royal United Hospital, Bath, on October 3rd and 4th. Five nurses presented themselves for examination, all of whom were successful in gaining the certificate of the hospital. The order of merit was as follows:—Nurse Edith Annie Hobbs, gold medallist; nurse Ada Maria Newell, silver medallist; nurse Lotty Mary Garratt, nurse Edith Annie George, nurse Ada Gayton. The examiners were Richard Scott, Esq., F.R.C.S., and Gilbert Bannatyne, Esq., M.D., the former senior surgeon, and the latter senior physician on the honorary staff of the hospital. The medals were the gift of the Rev. E. Handley, president of the hospital. The examination for second-year nurses was held on October 3rd and 8th, and proved most satisfactory. The order of merit was as follows:—Nurse Mildred Cope, nurse Cordelia Mackay, nurse Mabel Rees, nurse Elizabeth Williams, nurse Edith Chappell. The first and second nurses on the list were presented with medical books, the gift of the president's wife, Mrs. Handley.

### Appointments.

Central London Sick Asylum, Hendon.—Miss Agnes Hart has been appointed charge nurse. She was trained at the Toxteth Park Infirmary, Liverpool, where she has since been charge nurse.

Addenbrooke's Hospital, Cambridge.—Miss Morgan has been appointed matron. She was trained at the London Hospital, where she was subsequently sister. For the last six years she has been matron's assistant.

Chelmsford Union Infirmary.—Miss Christina Fulton has been appointed superintendent nurse. She was trained at Chester General Infirmary, and has since held the following posts: charge nurse, Birkenhead Union Infirmary; sister in the enteric wards, Bootle Corporation Hospital; district nurse, Nailsworth; superintendent nurse, Union Infirmary, Pontypridd.

District Hospital, Yeovil.—Miss Edith Blackler has been appointed staff nurse. She was trained at Taunton and Somerset Hospital, and has since been staff nurse at Rous Memorial Hospital, Newmarket; district nurse at Taunton; and charge nurse at Park Hospital, Lewisham. She has also been doing private nursing at Winchester.

Hull Sanatorium.—Miss W. L. Foster has been appointed sister. She was trained at the Hospital of St. Cross, Rugby.

Stockport Infirmary.—Miss Eleanor Richardson has been appointed matron. She was trained, for three years, at St. Bartholomew's Hospital, London. She has since been in charge of the Home Hospital, Eastbourne; sister of surgical wards and theatre at the Royal South Hants Hospital, Southampton; on the Army nursing staff at Wynberg and Bloemfontein in South Africa; and, from November, 1900, assistant matron at the Royal Hospital, Portsmouth.

St. George's Infirmary, Fulham Road, London.—Miss Elizabeth Rickards has been appointed charge nurse. She was trained at St. George's Infirmary, Fulham Road, and has since been staff nurse at Hove Hospital.

Tamworth Union Workhouse Infirmary. — Miss Caroline Richards has been appointed nurse. She was trained at University College Hospital and has since been nurse at the Dover Union Infirmary, and for four years on the staff of the Dover Nursing Institute.

Winchcomb Union Infirmary.—Miss Lizzie Pollard has been appointed assistant matron and nurse. She was trained at the Coventry and North Warwickshire Hospital, and was subsequently, for some years, staff nurse. She has since been doing private nursing.

Wolverhampton General Hospital.—Miss Philippa E. Martin has been appointed sister. She was trained at the General Infirmary, Cardiff, and has since been staff nurse at Birmingham General Hospital, and sister for two and a half years at the General Infirmary, Cardiff.

### Wants and Workers.

Hospital Trained Nurse (widow recently) would be so grateful for a disused warm long coat or cloak, too poor to purchase having three children and aged mother to support entirely. References gladly given. Address, Nurse, 1 Askew Crescent, Uxbridge Road, London, W.

### To Nurses.

We invite contributions from any of our readers, and shall be glad to pay for "Notes on News from the Nursing World," or for articles describing nursing experiences, or dealing with any nursing question from an original point of view. The minimum payment for contributions is 5s., but we welcome interesting contributions of a column, or a page, in length. It may be added that notices of entertainments, presentations, and deaths are not paid for, but, of course, we are always glad to receive them. All rejected manuscripts are returned in due course, and all payments for manuscripts used are made as early as possible after the beginning of each quarter.

### TRAVEL NOTES AND QUERIES.

Orotava and Teneriffe (Buffet).—Hotel accommodation is dear. The Grand Hotel, Orotava, charges from 10s. to 20s. per day. Hotel Martianez, that used to be the old Grand, is cheaper, 8s. to 10s. pension terms. Hotel Aguere and Continental, La Laguna, Teneriffe, about the same. The "English Grand Hotel," Port Orotava, Teneriffe, provides a nurse to invalids; one lives permanently in the hotel, which somewhat militates against an outsider setting up in the place. There are plenty of doctors of all nationalities, and the comfort of invalids is greatly studied. Did you want to know about nursing from a nurse's point of view or from a patient's? I should not think there was a good opening there for a nurse. There is a pension, "Sitio de Cullen," just outside Orotava, where, by arrangement, terms would be lower.

### Echoes from the Outside World.

AN OPEN LETTER TO A HOSPITAL NURSE.

The Duke and Duchess of Cornwall and York have gained a great deal of popularity in Canada by little gracious acts, which are the best possible indications of real kindness of heart. For instance, the Duke, on the occasion of his visit to Kingston, hearing that Dr. Grant, Principal of the University—who was recently made C.M.G. by His Royal Highness—was ill in bed in the General Hospital, insisted upon going to see him, and personally gave him the decoration. While the Heir Apparent was thus engaged, the Duchess sympathetically conversed with two sick nurses in the adjoining ward, expressing hopes for their speedy recovery. This is just what might have been expected from the Duchess of Teck's daughter.

A considerable success has been scored in South Africa. General French has captured Commandant Scheepers, though De Wet and Botha, more serious antagonists, are still not to be found. Commandant Lotter has, after trial, been executed for sedition, murdering scouts, murdering troopers in action on three occasions, blowing up railways and sjamboking British subjects. Two young farmers have also been hanged for having twice joined the enemy, and several other sentences of penal servitude for life, for ten years, or banishment from South Africa, have been passed. Sir Redvers Buller has caused a sensation by explaining in a speech at Westminster that, believing Sir George White had only enough food to last him a few days, he sent instructions in a cypher telegram at the end of 1899 to tell him his best way of surrendering. Fortunately, Sir George never needed to follow the instructions. Lady Methuen has just gone out to South Africa on a visit to her husband, who has now been out there two years, and the fact that she is joining him shows how little hope is entertained of his speedy return. Another steamer took out Lady French. Sir Elliott Lees, M.P., in addressing his constituents at Birkenhead the other day, was not afraid to enforce patience. He said he did not want to be pessimistic, but he should not be surprised if a year hence he were still asking them, as he did then, to be patient. To govern an Empire three things were wanted—blood, treasure and patience—and he thought that the nation meant to exercise the latter in spite of frothy and sensational journalism. If a few more men would speak in this admirable strain, perhaps there would be less twaddle in the papers about changing generals.

Although so much of summer is still left to us that we naturally refuse to don our warmer garments, it is not too early to begin to think about what we shall wear when the frost and the snow, or at least the damp and the cold, are all around us. First as to headgear. Everything is flat and round, no height is allowed anywhere, and except for the undulations caused by the introduction of a droop of lace, a tucked-in flower, or a beautiful buckle, the description I heard given by a schoolboy to his sister the other day was really very accurate. Much to his disgust, he had accompanied his mother to the wedding of a near relative, whilst his sister with a sprained ankle had remained at home. Upon his return she began laughingly to catechise him about the ladies' attire. "Oh, I don't know," was the rather irritable reply. "They were all howling swells, with trains which every fellow fell over, dresses which were so tight they looked as if they might crack, and dinner plates on their heads with a lot of stuff twisted straight round them." Full Tam-o'-Shanter crowns are to be seen everywhere, and they are a most convenient fashion for those whose purses are shallow. A hat with an unfashionable crown can often be made at once into one quite up-to-date by cutting away the centre of the hat, and supplying its place with a round stiffened piece of velvet with rows of fairly narrow velvet or ribbon run across and across in the same way as a jam tart is often decorated; this should be pleated on to the brim, and all raw edges covered with a twist of velvet, or a band of lace. Lace, by the way, is to play, if possible, a yet more important part this year in our winter costumes than it has done before. The most correct thing is, I hear, a muff, and a boa or pelerine both ornamented with beautiful old lace, and then the hat must unite both the fur and the lace in its composition so that all may be *en suite*. "Extremely nice, but also extremely dear," I daresay you will say, and I agree, though it is wonderful how much easier it is to work one decided scheme through a best "get-up" if only you think about it when first you begin to buy, rather than wait till several inharmonious items have been chosen, after which follows distress.

The impossibility of doing good work and keeping in good health without good food is happily impressing itself more and more upon the public mind. In one of the daily papers there is an able account by Miss Annesley Kenealy of the way the British Jack Tar is mis-fed, not for want of funds but for want of common sense. According to the victualling committee recently appointed to inquire into the rations supplied to the sailor "the fighting requirements of a battle-ship absorb the space which might otherwise be devoted to the refrigerating machinery necessary to the storage of fresh foods," which means in plain English that the man who does the fighting cannot, under existing conditions, be considered in at all the same proportion as the engine which drives the boat or the guns which fire the ammunition. As no refrigerator is allowed on any war-ship, neither butter nor cheese, fresh meat nor fruit, vegetables or milk are possible to help work the human machinery and keep it in thorough repair. And in this year of grace, 1901, tinned meat and the pickle-tub must be still the staple food of the man who joins the Royal Navy.

The other question is somewhat different. Miss O'Kell, sanitary inspector for Marylebone, draws attention to the need which exists for cheap restaurants to provide good meals for milliners, dressmaking hands, etc., whose wages are low. She shows that the want of nutritious food results in anæmia and kindred evils, and is most anxious that by philanthropic means it should be rendered possible to give a good meat meal for a sum which the women workers would be able to pay. Such, for instance, as can be purchased at Sir Thomas Lipton's great restaurant in the City Road, where portions of meat can be obtained for 1½d. or 2d., and a three-course dinner of soup, meat, and pudding for 4½d. But this is, of course, a charity pure and simple, and I must admit I have a strong sympathy with the lady who writes on the subject, to say that personally she is willing to house a girl, give her three good meals a day, two at least with meat, and 8s. or 10s. a week for dress, etc., but because the work she asks for is domestic service, with plenty of change of occupation, instead of long hours in crowded workrooms, she can obtain no assistance, and is expected as well to subscribe to funds to provide meals for the girls who, for the sake of "independence," prefer hard work and bad food. I do not suppose that even starvation will again induce the class from which domestic servants were originally gathered to return to housework, but at least self-supporting institutions should be possible, that they may be independent in something more than name.

In the *Lady's Gazette*—a new brightly written paper—which aims to give for the modest twopence much the same matter and illustrations as some of the other ladies' papers supply for sixpence, Madame Sarah Grand ventilates her views upon things in general. She leans towards woman's suffrage, and though she does not see why a woman need necessarily sit in Parliament because she has a vote, fails to recognise anything more awful in the prospect of a House of Ladies than in a House of Lords. She points out that if women were not a success in Parliament their constituents would naturally not elect them again, and that therefore little harm would be done. On the subject of vivisection Madame Grand is very outspoken and very antagonistic. The form of the chat has the merit of originality. It is a three-sided affair. Madame Grand talks, the interviewer asks questions, and the "looker on" writes down the remarks of both. I do not think the plan would be always satisfactory. A timid man suddenly confronted by two lady interviewers might possibly be too nervous to be communicative; but perhaps the interview *à trois* is only to be resorted to occasionally.

### For Reading to the Sick.

"HE LEADETH ME."

He knows His sheep;

He counts them, and He calleth them by name.

He goes before;

They follow as He leads, through flood or flame.

He leads them out
Into the pastures green, by waters still;
He leads them in,
And guards them safe, within the fold, from ill.

And when this day
Of storm and scattering is ended here,
Thou wilt them bring
To greener pastures, and to streams more clear.

*H. Bonar, D.D.*

Another note which rings out clearly in this verse is peace. "He maketh me to lie down . . . He leadeth me.' How sadly the soul needs peace—peace in His felt presence! The world is sown with trouble, but still "He maketh me to lie down . . . He leadeth me."

These green pastures are no luxury of religion; they are a necessity of life. Each day must have its Nazareth of devotion, as life has its own Nazareth of subjection in childhood; times of refreshment, when He maketh me lie down in the green pastures of living rest. Let us cheerfully recognise that, if the good Shepherd is leading us, there is no such thing as accident. Trifles may very easily interfere with our peace of mind; but they may also be God's messengers to teach us to cast away all appearance of grumbling and fretfulness. As we pass along our way, led by the good Shepherd in the repose of the green pastures, in the peace of the abiding Presence, in the comfort of the still waters, we can say once more, with a new depth of meaning, "Here is the guardianship of the peace of God, which 'passeth all understanding.'"—*Canon Newbolt.*

Every nook of the mountain, every grassy knoll,—ay, too, and every bleak corner of these pasture grounds—are known to Him. As an old writer quaintly says, "He leads us in, He leads us through, He leads us on, He leads us up, He leads us home!"—*J. R. Macduff, D.D.*

Peace have I left with you, My peace have given,
Not as the world doth give,
But as cool balm upon a spirit riven,
Soft air where billows strive,
Or the blue widening gleam that parts the stormy heaven.

If thou wilt hear Me, and wilt make thy choice
To follow where I lead,
As one who knoweth well his Shepherd's voice,
And loves the sheltered mead,
Then in fair peace shall all thy heart rejoice;

Then thou shalt find it in the meadow wide,
Where whitest flocks are fed;
In pastures green with it shalt thou abide,
By living waters led;
With it from noon-day heat in deepest shadows hide.

*From the "Inner Life."*

### Notes and Queries.

**The Editor is always willing to answer in this column, without any fee, all reasonable questions, as soon as possible.**
**But the following rules must be carefully observed:—**
**1. Every communication must be accompanied by the name and address of the writer.**
**2. The question must always bear upon nursing, directly or indirectly.**
**If an answer is required by letter a fee of half-a-crown must be enclosed with the note containing the inquiry.**

*Disinfecting Money.*

(24) I should be glad if you could tell me a quick way of disinfecting money. Our funds are kept up by the poor, paying 1d. weekly, which has to be collected by a collector. Lately there have been some cases of scarlet fever break out, and our Secretary objects to take the money till it has been disinfected. As it is mostly copper coin, I am puzzled what to do. I should be much obliged if you could give me a hint as to the best way.—*District Nurse.*

Put it in a pan and boil it.

*"Whitehead and Circumcision."*

(25) Will you tell me if the action of bowels is usually interfered with after the operation called "Whitehead"? What is the operation beyond removing piles? Why is circumcision performed upon healthy babes apart from Jewish law?—*A St. Thomas's Nurse.*

The essence of Whitehead's operation lies in the fact that instead of each individual pile being removed separately the whole of the "pile area" is taken away, the edge of the mucous membrane above being attached to the anal margin. Those who oppose this operation hold that it may in certain cases lead to stricture from the contraction of the circular ring of scar tissue, while those who support it maintain that there is no such risk. Probably a good deal depends upon how it is done. If healing should not take place at once no doubt there would be left a ring of scar tissue which might perchance contract, but if immediate union should occur there would not be this risk. In regard to circumcision, there are many advantages in having it done whenever there is any difficulty in retracting the foreskin. A foreskin which cannot be retracted forms a bag which becomes filled with much foul material, and this in turn sets up irritations and abnormal sensations which are the root of much evil.

*Opening for Private Work.*

(26) I am anxious to go to the Cape to take up private nursing. Will you kindly tell me (1) where the best opening would be, and (2) the cost of the passage out?—*E. R. I.*

1. Write to the Emigration Information Office, 31 Broadway, Westminster, S.W., or to the Hon. Sec., the United British Women's Emigration Association, Imperial Institute, S.W., for latest official returns. 2. Second class passage, £24 3s.

*Abroad.*

(27) Can you tell me where I can get information on the subject of nursing on the Riviera? Salary, climate, work, etc.—*Miss H.*

The Lady Superintendent, the Nice Nursing Institute, Villa Soleil, Avenue Thiers, Nice, could give full information, or Miss Bryant, Matron of English Nurses' Institutes at San Remo, Bordighera and Alassio.

*Incurables.*

(28) Can you inform me how patients gain admittance into the various hospitals for incurables?—*E. S.*

Generally by votes. Apply, asking for rules of procedure, to the secretary of the hospital you wish to enter.

*Homes.*

(29) I have a pleasant home where I receive poor ladies at reduced fees. Will you kindly tell me if I can answer suitable inquiries in your Notes and Queries column, and how to do so.—*I. J. J.*

Enclose your reply in an envelope bearing the number, the heading, and the initials of query, and a stamp. Send it to this office, and it will be forwarded.

**Standard Books of Reference.**

"The Nursing Profession: How and Where to Train." 2s. net; post free 2s. 4d.
"Burdett's Official Nursing Directory." 3s. net; post free, 3s. 4d.
"Burdett's Hospitals and Charities." 5s.
"The Nurses' Dictionary of Medical Terms." 2s.
"Burdett's Series of Nursing Text-Books." 1s. each.
"A Handbook for Nurses." (Illustrated). 5s.
"Nursing: Its Theory and Practice." New Edition. 3s. 6d.
"Helps in Sickness and to Health." Fifteenth Thousand. 5s.
"The Physiological Feeding of Infants." 1s.
"The Physiological Nursery Chart." 1s.; post free, 1s. 3d.
"Hospital Expenditure: The Commissariat." 2s. 6d.
All these are published by the Scientific Press, Ltd., and may be obtained through any bookseller or direct from the publishers 28 and 29 Southampton Street, London, W.C.

## Figures and Tables

**Fig. 2. f1:**
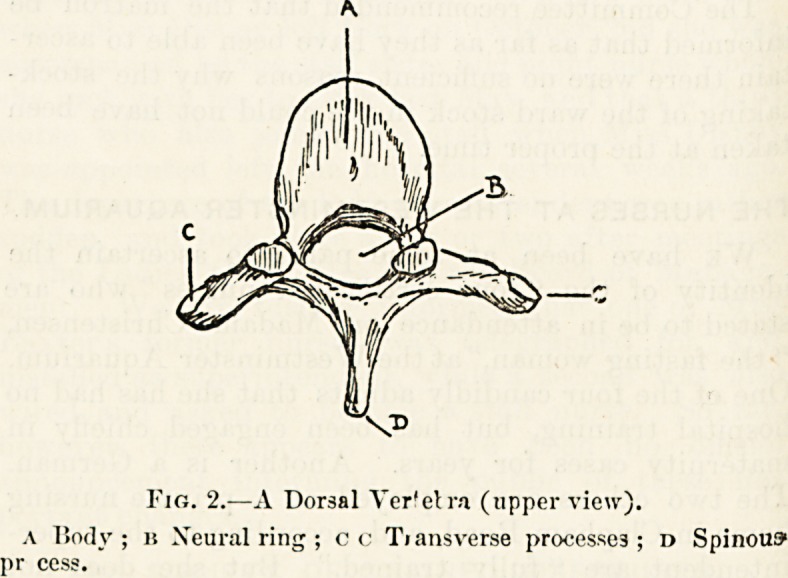


**Fig. 3. f2:**